# Dual Sensory Impairment and Cognitive Performance, MCI, and Dementia in Older Adults: The ARIC Neurocognitive Study

**DOI:** 10.1093/geroni/igaa057.2925

**Published:** 2020-12-16

**Authors:** Jennifer Deal, Junghyun Park, Nicholas Reed, Alison Abraham, Frank Lin, B Gwen Windham, A Richey Sharrett

**Affiliations:** 1 Johns Hopkins Bloomberg School of Public Health, Baltimore, Maryland, United States; 2 New York University, New York City, New York, United States; 3 Johns Hopkins University, Baltimore, Maryland, United States; 4 Johns Hopkins University School of Medicine, Baltimore, Maryland, United States; 5 University of Mississippi Medical Center, Jackson, Mississippi, United States

## Abstract

Dual sensory impairment (DSI) affects 11.3% of adults aged ≥80 years. Hearing and vision impairments are each associated with cognitive decline and dementia, but DSI’s impact is unknown. All-cause dementia and mild cognitive impairment (MCI) were adjudicated using longitudinal cognitive information. Ten neurocognitive tests were summarized using latent variable methods. Hearing was measured using pure tone better-ear thresholds (0.5-4 kHz) and vision with better-eye presenting distance visual acuity and/or contrast sensitivity. In 881 adults (79±4 years, 44% black, 64% female), DSI (vs. no hearing or vision impairment) was cross-sectionally associated with -0.17 standard deviations (SD) [95% confidence interval (CI): -0.32, -0.02] lower global cognitive score and an 87% increased odds (95% CI: 1.01, 3.45) of combined MCI/dementia, after full adjustment for demographic and clinical factors. Future longitudinal research should elucidate the mechanism underlying this association to determine if treatment can delay cognitive decline and MCI/dementia in older adults.

